# A novel therapeutic concern: Antibiotic resistance genes in common chronic diseases

**DOI:** 10.3389/fmicb.2022.1037389

**Published:** 2022-10-17

**Authors:** Xiaxia Pan, Ziyuan Zhou, Bowen Liu, Zhongwen Wu

**Affiliations:** State Key Laboratory for Diagnosis and Treatment of Infectious Diseases, National Clinical Research Center for Infectious Diseases, National Medical Center for Infectious Diseases, Collaborative Innovation Center for Diagnosis and Treatment of Infectious Diseases, The First Affiliated Hospital, Zhejiang University School of Medicine, Hangzhou, China

**Keywords:** gut microbiota, antibiotic resistance genes, horizontal gene transfer, chronic diseases, monitoring and treatment

## Abstract

Infections caused by multidrug-resistant bacteria carrying antibiotic resistance genes pose a severe threat to global public health and human health. In clinical practice, it has been found that human gut microbiota act as a “reservoir” of antibiotic resistance genes (ARGs) since gut microbiota contain a wide variety of ARGs, and that the structure of the gut microbiome is influenced by the profile of the drug resistance genes present. In addition, ARGs can spread within and between species of the gut microbiome in multiple ways. To better understand gut microbiota ARGs and their effects on patients with chronic diseases, this article reviews the generation of ARGs, common vectors that transmit ARGs, the characteristics of gut microbiota ARGs in common chronic diseases, their impact on prognosis, the current state of treatment for ARGs, and what should be addressed in future research.

## Introduction

The development and dissemination of antibiotic resistance genes (ARGs), the genetic basis of drug-resistant bacterial strains, are complex. Drug resistance is associated with the existence of genes in the bacterial genome that can produce drug-resistant phenotypes. And different genera, species and strains can exhibit different antibiotic-resistant phenotypes (Davies and Davies, 2010). The prevalence, categories, and resistance mechanisms of ARGs vary geographically and between body sites (Carr et al., 2020). The composition of intestinal ARGs is affected by many factors, and antibiotic use has a significant effect on the composition of gut microbiota and the profile of ARGs therein. Besides, antibiotic drugs can induce the development of ARGs and promote their transmission as well (Nobel et al., 2015; Jutkina et al., 2016; Doan et al., 2020).

The gene transmission between individuals except the parent-offspring relationship is a common phenomenon and acts as an important source of genetic diversity which called the horizontal gene transfer (HGT) (Soucy et al., 2015). The human gut microbiome harbors a large number of ARGs, which can spread rapidly among bacterial pathogens through HGT (Penders et al., 2013; McInnes et al., 2020). The spread of antibiotic resistance poses a serious risk to human health. The effects of gut microbiota and ARGs in common chronic diseases, such as liver cirrhosis (Shamsaddini et al., 2021), diabetes (Shuai et al., 2022), and chronic kidney disease (CKD) (Wang X. et al., 2020) have been investigated, characteristic alterations of ARGs in different disease states have been highlighted, and there is an increasing abundance of ARGs with disease progression.

In this review, we searched PubMed and Web of Science with keywords such as antibiotic resistance genes, chronic diseases, gut microbiome and horizontal gene transfer. We summarize the latest studies which identify gut microbiota and ARGs in common chronic diseases such as liver cirrhosis, diabetes, and CKD to discuss the role and characteristic of ARGs in the progression of related diseases. This manuscript should provide new concern for the development of individualized treatments targeting the monitoring and management of gut ARGs in common chronic disease.

## Effects of drugs on the emergence, expansion and transmission of gut antibiotic resistance genes

Gut microbiota have gained increasing attention as a reservoir for ARGs, which can be transferred within the gut microbiota as well as to certain bacteria that simply pass through the gut (Hu et al., 2013; Penders et al., 2013). The factors that contribute to ARG development and prevalence are complex. It is well known that the exogenous substances such as antibiotic drugs have a notable effect on ARGs, and some commonly used non-antibiotic drugs may also alter the constitution of gut microbiota and the transmission of ARGs (Hu et al., 2013; Maurice et al., 2013).

### Effect of antibiotic drugs on the emergence, expansion and transmission of antibiotic resistance genes

Gut microbiota are frequently exposed to antibiotics, which is the key driver of bacterial antibiotic-resistance and leads to the prolonged presence of ARGs in the gut microbiota (Founou et al., 2016). Antibiotic-induced alteration of the gut microbiota directly causes a shift in the profile of resistance genes including increasing mutations, expression and transmission.

Doan et al. investigated the gut resistome in over 500 children who used azithromycin twice a year for 4 years, and found that large-scale use of azithromycin may induce an increase abundance of ARGs (Doan et al., 2020). A study in which cefprozil was administered to healthy volunteers found that standard antibiotic therapy changes the gut microbiota specifically and predictably according to the initial gut microbiota composition which further alters the abundance of ARGs. For example, oral antibiotic treatment led to an increase in point mutations in the β-lactamase resistance gene blaCfxA-6 and an increasing abundance of some conditionally pathogenic bacteria, such as Enterobacter cloacae which may suggest that pre-treatment monitoring of gut microbiota composition can help avoid adverse effects of antibiotic therapy (Raymond et al., 2016). Another study also found that antibiotic-specific resistance gene homologs (AsRGs) had high transcriptional activity during antibiotic therapy and the relative abundance sustained expansion for 3 months which suggested a long-lasting increment of ARGs expression (Kang et al., 2021). Additionally, Nobel et al. (2015) established a mouse model simulating pediatric antibiotic use and found that the two widely used classes of antibiotics including beta-lactam and macrolide, had profound effects on the gut microbiome and metagenome. Early therapeutic-dose pulsed macrolide treatment (PAT) induced alterations in the mouse gut microbiota and increased the expression of four ARGs (acrA, acrB, ant3Ia, and ant2Ia) associated with macrolide-resistance. Except increase the mutations or expression of ARGs, antibiotic drugs can also promote their transmission. Jutkina et al. found that very low concentrations (10 μg/L) of tetracycline drive the transfer of diverse ARGs (Jutkina et al., 2016). Wu et al. discovered that levofloxacin induced the plasmid mediated transformation and increased both the abundance and spread of antibiotic-resistant Escherichia coli (Wu H. Y. et al., 2020). Understanding the role of antibiotics in regulating the expression and propagation of ARGs is critical.

### Effect of non-antibiotic drugs on the emergence, expansion and transmission of antibiotic resistance genes

Some commonly used non-antibiotic drugs can influence the gut microbiota composition, which can manifest as a high incidence of antibiotic-like side effects. About 24% of non-antibiotic drugs targeting humans which inhibit the growth of at least one gut bacterial strain could promote antibiotic resistance (Maier et al., 2018). Non-antibiotic drugs use may be a causal factor in the generation and spread of intestinal ARGs. Wang et al. found that five non-antibiotic drugs including anti-inflammatory drugs (ibuprofen, naproxen, and diclofenac), lipid-lowering drugs (gemfibrozil), and β-blockers (propranolol) promoted transmission of ARGs through bacterial transformation (Wang Y. et al., 2020).

## Common vectors of antibiotic resistance genes transmission in human gut

**FIGURE 1:**
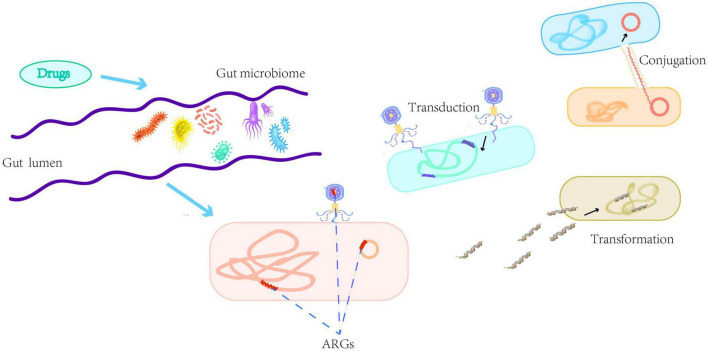
A brief description of the emergence of antibiotic resistance genes (ARGs) and the main horizontal gene transfer modes of ARGs in the gut including transformation, transduction, and conjugation. Transformation refers to the taking up of naked deoxyribonucleic acid from the environment by physiologically competent bacteria. Transduction refers to the transfer of phages carrying genetic material between donor and recipient bacteria. Conjugation refers to mobile genetic elements, such as plasmids, transferring through a pilus formed between the donor and recipient cells.

Although bacterial communities in human and animal gastrointestinal tracts, rivers, and wastewater are major sources of ARGs, the origin of these ARGs and the process of transmission from the environment to clinical settings are still poorly understood (Larsson and Flach, 2021; Li et al., 2021; Zhuang et al., 2021). The spread of ARGs in the human gut is more complex where ARGs can be transmitted through HGT (including transformation, transduction, and conjugation) (McInnes et al., 2020). The extensive spread of ARGs, due to antibiotic abuse, is associated with different vectors including the plasmids, viruses and bacteria that carry ARGs and act as carriers to transfer genetic information between microbial species (Botelho et al., 2019; McInnes et al., 2020). We describe the emergence of ARGs and the main HGT modes of ARGs in the gut in Figure 1.

### Plasmids; the major vector

Plasmids are autonomously replicating DNA molecules which can carry multiple resistance genes and are the main mode of transmission of ARGs between bacteria. Plasmid-based HGT plays an important role in the spread of ARGs between bacterial conspecifics or different species, and the evolutionary status of plasmid-carrying bacteria is also an important factor in the transmission of antibiotic resistance plasmids between bacterial pathogens *in vivo* (Navon-Venezia et al., 2017; San Millan, 2018; Stalder et al., 2019). Gumpert et al. provided the first genomic-level characterization of ARG transfer in undisturbed human gut microbiota and demonstrated that plasmids carrying ARGs, in the infant, persist and are transferred even in the absence of antibiotic administration (Gumpert et al., 2017). Besides, it has been proposed that establishing linkage between plasmids and hosts in wastewater environments, through the *in vivo* proximity-ligation method, can accurately identify carriers of ARGs and help limit the spread of pathogen resistance (Stalder et al., 2019).

### Viruses; a key vector

Viruses are also thought to be key vectors for ARG transfer, enabling the spread of ARGs more broadly than in bacterial genomes. ARGs have been observed only in a very small number of phages, through virome sequencing and assembly analysis, but phages carrying ARGs are known to exist in the human gut and other environments. Phage-mediated HGT diversifies the microbial gene pool, and since phages can survive in the environment for a long time, they can prolong or delay the transfer of genetic information (Touchon et al., 2017; Debroas and Siguret, 2019). Antibiotic treatment may contribute to the release of bacterial prophages carrying ARGs, leading to an increase in free phages carrying ARGs in the human gut, thereby spreading drug resistance (Fernandez-Orth et al., 2019). AsRGs are frequently present on MGEs, and the relative abundance of mobile AsRGs is often remains consistently high even 3 months after antibiotic treatment. Consequently, MGEs contribute to the expansion and persistence of antibiotic resistance gene pools, and mobile AsRGs also enable transfer of antibiotic resistance through potential HGT (Kang et al., 2021).

### Bacteria play a role in the spread of antibiotic resistance genes

Bacteria in the gut not only acquire ARGs, but also contribute to the transfer of ARGs to other bacteria in the gut (Ravi et al., 2014). Jiang et al. demonstrated that ARGs can be transferred from antibiotic-producing actinobacteria to proteobacteria, which may be related to conjugative transfer of a carrier sequence from proteobacteria to actinobacteria (Jiang X. et al., 2017). As early as 1988, quinolone resistant strains of Klebsiella pneumoniae were found that could transmit low level of quinolone resistance to other bacteria (Martínez-Martínez et al., 1998). K. pneumoniae is a major source of global drug resistance with ARGs located on both the chromosome and plasmids of the bacterium. Under antibiotic selection pressure, the bacterium continues to accumulate ARGs through *de novo* mutations and MGEs, eventually resulting in the appearance of super-resistant strains. ARGs continue to accumulate as the resistome evolves, and K. pneumoniae strains have been shown to harbor 52 antibiotic resistance plasmids capable of promoting the transfer of resistance through HGT (Navon-Venezia et al., 2017). Pseudomonas aeruginosa can also acquire drug resistance through chromosomal mutations and acquisition of ARGs by HGT (Botelho et al., 2019). Salmonella enterica serovar Typhimurium (S.Tm) can survive in host tissues during antibiotic treatment, and reimplantation of these bacteria into the intestinal lumen facilitates the transfer of plasmids carrying ARGs between different Enterobacteriaceae. Small reservoirs of persistent pathogens can also encourage the spread of mixed resistance plasmids among microbiota in the gut even in the absence of selection for resistance genes encoded by plasmids (Bakkeren et al., 2019). Moreover, (Klassert et al., 2021) observed a dynamic process of bacterial colonization and spread of ARGs after initial patient admission and sequencing data revealed that a stable community structure formed at three indoor sites in the hospital environment (floor, doorhandle and sink) in only a few weeks, and that the rapid colonization process of microbiota correlated with a remarkable increase in ARGs. Studying HGT in the gut microbiota could help to identify and develop new interventions aimed at minimizing the transmission of ARGs between commensals and opportunistic pathogens.

## Antibiotic resistance genes in common chronic diseases

Currently, the presence of chronic diseases is now a major challenge to global health,representing a diverse group of diseases that last a year or more and progress slowly. Chronic disease include, but is not limited to, a variety of disorders such as diabetes, cardiovascular disease (CVD), chronic kidney disease (CKD) or chronic liver disease, but could also involve some chronic infections (Bauer et al., 2014; Bergman and Brighenti, 2020). In the long-term clinical progression, those patients with chronic diseases are more vulnerable to various infection and thus, are more likely to be exposed to different antibiotics. However, as discussed above, the emergence of antibiotic resistance affects community structure, and alterations in gut microbiota, caused by antibiotic exposure, are associated with certain metabolic diseases (Blaser and Falkow, 2009; Blaser, 2016; Joossens et al., 2019).

A previous study analyzing the ARGs of gut microbiota from 162 people found a total of 1,093 ARGs. Analysis of the microbial origin of the ARGs revealed that they were more likely to be found in Proteobacteria, and regional differences in ARGs revealed different ratios of resistance genes to total intestinal genes in Chinese, Danish and Spanish populations, with ratios of 0.94, 0.44, and 0.89%, respectively. The Chinese population has a higher abundance of resistance genes and a greater variety of resistance gene types, with tetQ and ermB being the two most abundant ARGs (Hu et al., 2013). Yang et al. (Yang et al., 2016) performed a preliminary analysis of 1,267 stool samples from 368 Chinese, 139 American, 401 Danish and 359 Spanish, to investigate country-specific gut resistance profiles, and found a total of 112 ARGs were present in 90% of the samples from different countries, with the highest abundance of ARGs detected in samples from China, and the duration of use and applicability of antibiotics were correlated with the accumulation of ARGs in gut microbiota.

**FIGURE 2:**
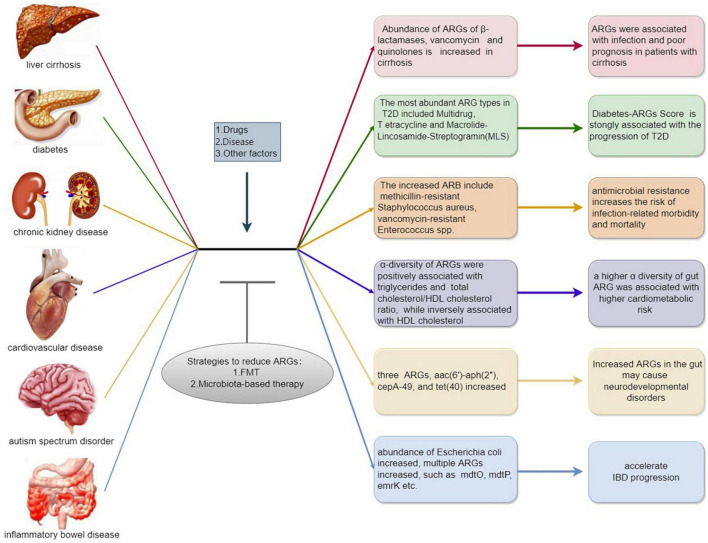
A brief introduction of antibiotic resistance genes (ARGs) in common chronic diseases. Exogenous and endogenous stimulation such as drugs and chronic diseases are positively correlated to the emergence and expansion of ARGs, which may conversely aggravate the progression of chronic diseases. While microbial intervention including fecal microbiota transplant, prebiotic and probiotic exhibit the potential to downregulate the abundance of ARGs which may improve related diseases.

The state of diseases can also influence ARGs, and microbial species specifically related to certain diseases may be hosts for ARGs. For example, Escherichia coli carries multiple ARG subtypes, so monitoring ARGs in this bacterium can help predict or treat certain diseases (Qiu et al., 2020). A brief introduction of ARGs in common chronic diseases can be seen in Figure 2.

### Antibiotic resistance genes in cirrhosis

Patients with cirrhosis are prone to infections and this is may be related to alterations in the composition of gut microbiota in these patients that make them more susceptible to colonization by antibiotic-resistant bacteria and pathogenic species (Bajaj et al., 2019a,b; Tranah et al., 2021). Previous research has demonstrated that progressive changes in gut microbiota of patients with cirrhosis are strongly associated with disease progression and poor prognosis (Bajaj et al., 2014; Sung et al., 2019; Sole et al., 2021; Trebicka et al., 2021). The abundance of pathogens that express ARGs increases as cirrhosis progresses (Bajaj et al., 2014; Qin et al., 2014; Chen et al., 2015). Thus, detecting and targeting ARGs may improve the clinical outcomes of patients with cirrhosis. Shamsaddini et al. (2021) investigated characteristic ARGs that correlated with infection and poor outcomes, in the gut microbiota of patients with cirrhosis. Pathogenic-related resisitomes, such as Enterobacteriaceae, Streptococcus spp., Enterococcus spp. and Fusobacterium spp., were more abundant in cirrhotic patients than in healthy controls, resulting in a higher abundance of ARGs related to β-lactamases, vancomycin and quinolones, in the gut of cirrhotic patients, with disease progression. ARGs associated with hepatic encephalopathy (HE) and ascites have also been identified.

Patients with HE had a higher abundance of ARGs associated with β-lactamases, vancomycin resistance and RbpA bacterial RNA polymerase binding protein, than non-HE patients, while quinolone resistance genes were lower than those in non HE patients. This study also found that ascites was connected with an increase in Pseudomonas, Serratia and Clostridium perfringens in the gut, while PPI use was related to higher vancomycin resistance. PPIs can modulate the composition of the gut microbiota in cirrhotic patients, and systematic stopping and judicious use of PPIs is needed in patients with decompensated cirrhosis (Bajaj et al., 2018; Shamsaddini et al., 2021).

ARG abundance increases in Gram-positive bacteria in cirrhosis patients, and these ARGs differ from those seen in type 2 diabetes (T2D) and CKD patients. Cirrhosis was significantly separated from both diabetes and CKD by principal coordinate analysis, and six ARGs differed between the cirrhosis and diabetes groups. Patients with cirrhosis had a greater abundance of ARGs, with a broader resistance spectrum. These ARGs belonged to a variety of pathogenic Gram-positive and Gram-negative bacteria (Qin et al., 2012; Shamsaddini et al., 2021).

A prospective assessment of two groups of European patients with decompensated cirrhosis, found that infections caused by antibiotic-resistant bacteria were common in cirrhosis patients. Among culture-positive infections, those caused by multidrug-resistant microorganisms accounted for 29% in the year 2011 and 38% in 2017–2018, with the most common multidrug-resistant bacteria being ultra-broad-spectrum β-lactamase-producing Enterobacteriaceae. The study also found that antibiotic resistance patterns were highly heterogeneous, with significant variation in multidrug-resistant bacteria types acrossed countries and centers (Fernandez et al., 2019). In European patients with severe liver disease, multidrug-resistant bacterial infections have become a common and growing medical problem associated with poor prognosis, and preventing the spread of antibiotic resistance in cirrhosis is critical.

### Antibiotic resistance genes in diabetes

Previous studies have shown that gut microbiota composition is strongly associated with T2D and that microbiota in T2D patients change in line with blood glucose levels (Qin et al., 2012; Brunkwall and Orho-Melander, 2017; Wu H. et al., 2020). Antibiotic use disrupts gut microbiota composition and long-term use can increase the risk of T2D in women (Yuan et al., 2020). The study of a large human cohort revealed the detailed profiles of the gut antibiotic resistome in different disease conditions, revealing a new association between the resistome, gut microbiota, and the progression of T2D (Shuai et al., 2022). The most enriched ARGs in this cohort included multidrug, tetracycline and Macrolide-Lincosamide-Streptogramin (MLS), with tetracycline tetQ being the most abundant ARGs subtype, which is more in line with previous study (Hu et al., 2013; Shuai et al., 2022). Due to the overall shift in gut ARGs and microbiota composition in the healthy, prediabetes, and T2D groups, a new Diabetes-ARGs Score (DAS) was proposed. The DAS is strongly associated with the progression of T2D and gut ARGs can be found in a wider range of bacterial species as the disease progresses, which may explain some of the pathophysiological mechanisms associated with T2D. There were 25 ARGs associated with T2D, and genome-wide association analysis of selected ARGs revealed that Vancomycin_vanX, Multidrug_emrE, MLS_ermX, and Quinolone_norB were all positively correlated with a high risk of T2D, and that the abundance of Quinolone_norB and β-lactam subtypes increased with diabetes progression. Genetic tools were constructed for these ARG profiles, and genetic predictions also confirmed that the higher abundance of intestinal ARGs is associated with a high risk of T2D. It was also found that changes in gut antibiotic resistance preceded changes in gut microbiota during T2D progression and the pattern of ARG abundance shifted as disease progressed. Intestinal antibiotic resistance was also associated with fecal metabolites; for example, DAS and Vancomycin_vanX were positively correlated with L-isoleucine and L-leucine in fecal samples (Wu H. et al., 2020; Shuai et al., 2022). The above confirmed the significant association of plasma L-isoleucine and L-leucine with future diabetes risk seen in a previous study (Wang et al., 2011). These results reveal a new horizon between gut microbiota and T2D progression, which was revealed by studying antibiotic resistance, and these characteristic ARGs may become targets for T2D intervention in future studies.

### Antibiotic resistance genes in chronic kidney disease

Gut microbiota composition is remarkably altered in patients with CKD, and this is related to the inflammatory state and renal function in CKD patients (Vaziri et al., 2013; Jiang S. et al., 2017; Xu et al., 2017; Meijers et al., 2019). Since patients with CKD, especially dialysis patients, are susceptible to infections they are considered to be reservoirs of antibiotic-resistant pathogens (Su et al., 2018). Antibiotic-resistant bacteria (ARB), including methicillin-resistant Staphylococcus aureus, vancomycin-resistant Enterococcus spp., and several multidrug-resistant gram-negative organisms, colonize and infect patients with CKD who require dialysis or kidney transplantation. Moreover, increased antibiotic resistance is positively associated with the risk of infection and death. Consequently, clinicians must be familiar with the local epidemiological history associated with ARBs and remain alert to the emergence of new resistance patterns (Zacharioudakis et al., 2015; Wang et al., 2019). Imbalance in gut microbiota can promote further deterioration of renal function and lead even to renal failure. Animal models of CKD have demonstrated that two microorganisms, Eggerthella lenta and Fusobacterium nucleatum, can produce serum uremic toxins and exacerbate the development of kidney disease. The prevalence of several other pathogenic bacteria such as Klebsiella pneumoniae, Acinetobacter, Enterobacter, and Legionella, also increases during CKD (Wang X. et al., 2020). When comparing CKD and cirrhosis, only seven ARGs were found to differ between the cirrhosis and CKD groups of patients, and β-lactamase genes were present in both disease groups. The abundance of glycopeptide, vancomycin, cephalosporin, and rifamycin resistance genes was higher in patients with cirrhosis, and the abundance of ARB belonging to a wide spectrum of Gram-negative and Gram-positive pathogenic microorganisms is also increased in patients with CKD (Wang X. et al., 2020; Shamsaddini et al., 2021). The abundance of ARGs is higher in patients with cirrhosis than in those with CKD, and the more highly abundant ARGs are associated with high antibiotic use and increased abundance of antibiotic-resistant pathogens (Su et al., 2018; Shamsaddini et al., 2021). Patients with kidney impairment are at high risk of infections caused by multidrug resistance organisms, and kidney dysfunction at the time of admission should be used as a signal to closely monitor microbiological culture results thereafter (Su et al., 2018). It has been shown that the gut microbiome and the expression of intestinal genes are both altered in patients with CKD. Thus, increases in Proteobacteria at the phylum level, Enterobacteriaceae and Corynebacteriaceae at the family level, Enterococcus and Clostridium at the genus level are all characteristically observed in CKD patients, and the expression of genes related to the trimethylamine (TMA) metabolic pathway are altered in CKD. TMA is absorbed by the liver and oxidized to trimethylamine N-oxide (TMAO), which is ultimately excreted by the kidney. Impaired kidney function and an imbalance of gut microbiota in CKD patients causes elevated plasma TMAO, which increase cardiovascular risk in patients with CKD (Xu et al., 2017).

### Antibiotic resistance genes in cardiovascular disease

Alterations in the gut microbiota are also associated with the development of cardiovascular disease. Several members of the Enterobacteriaceae, including Escherichia coli, Klebsiella spp., and Enterobacter aerogenes and oral bacteria such as Streptococcus spp. and Lactobacillus salivarius, are all more abundant in patients with atherosclerotic cardiovascular disease (CVD), whereas the levels of butyric acid-producing bacteria, such as Roseburia intestinalis and Faecalibacterium cf. prausnitzii, are reduced (Jie et al., 2017; Tang et al., 2017; Witkowski et al., 2020). Shuai et al. (2022) also observed that differences in gut antibiotic resistance are associated with cardiometabolic risk; the α-diversity indices of gut ARG were strongly correlated with triglycerides and the total cholesterol/high-density lipoprotein (HDL) cholesterol ratio but negatively associated with HDL cholesterol. Further analysis confirmed that these correlations were unrelated to T2D and that a higher α diversity of gut ARG was linked to higher cardiometabolic risk. Yuan et al. (2020) proposed that prolonged antibiotic use was associated with future cardiovascular disease incidence. Identifying new therapeutic options for altered gut microbiota composition could provide a new direction for future treatments. There are eight species in the gut, including Anaerococcus hydrogenalis, Clostridium asparagiforme, Clostridium hathewayi, that are associated with TMA accumulation (Romano et al., 2015). Elevated plasma TMAO levels can promote the acceleration of chronic diseases including CVD, T2D, and CKD through a variety of mechanisms. TMAO also promotes oxidative stress in blood vessels and dysfunction of the endothelium. Consequently, the inhibition of TMAO production, a gut microbiota metabolite, is a new strategy for the management of several common chronic diseases (Mente et al., 2015; Wang et al., 2015; Chen et al., 2019; Brunt et al., 2020).

### Antibiotic resistance genes in other chronic diseases

ARGs in gut microbiome can affect brain function and significant changes in the gut microbiota of children may cause neurodevelopmental disorders. Kovtun et al. studied the distribution of ARGs in the gut microbiome of 3–5 years old healthy children and children with autism spectrum disorder (ASD) by metagenomic analysis. They found significant differences characterized by increased ARGs in the gut microbiome of children with ASD. Genes aac(6′)-aph(2″) from Enterococcus, cepA-49 from Bacteroides (b-lactams) and tet(40) from Megasphaera, are three specific ARGs in ASD (Kovtun et al., 2020). Another study found significant changes in the gut microbiota in patients with inflammatory bowel disease (IBD). Increased abundance of pathogenic Escherichia coli showed a positive correlation with multiple ARGs, such as mdtO, mdtP, emrK, etc., which may accelerate IBD progression. For ARG types, beta-lactam, fosmidomycin, multidrug and polymyxin resistance genes were significantly enriched in Crohn’s disease (CD) patients (Xia et al., 2021). Duvallet et al. (2017) found that 10–15 genus-level changes among gut flora could lead to the development and progression of multiple human diseases. The close relationship between ARGs and chronic diseases suggests potential value to monitor and evaluate gut microbiota and the ARGs of patients with chronic diseases. The variation of ARGs in common chronic diseases and their impact on disease development are listed in Table 1.

**TABLE 1:** The variation of antibiotic resistance genes (ARGs) in common chronic diseases and their impact on disease development.

Types	Variation	Impact	References
Cirrhosis	ARGs related to β-lactamases, vancomycin, quinolones increase	Infection and poor outcomes	Shamsaddini et al., 2021
HE	Higher abundance of ARGs associated with β-lactamases, vancomycin resistance and RbpA bacterial RNA polymerase binding protein	Disease progression	Shamsaddini et al., 2021
Ascites	Pseudomonas, Serratia and Clostridium perfringens increase	Disease progression	Shamsaddini et al., 2021
Diabetes	25 ARGs associated with T2D, including Vancomycin_vanX, Multidrug_emrE, MLS_ermX and Quinolone_norB	High risk of T2D	Shuai et al., 2022
	DAS and Vancomycin_vanX, positively correlated with L-isoleucine and L-leucine	Future diabetes risk	Wu H. et al., 2020; Shuai et al., 2022; Wang et al., 2011
CKD	ARB have high prevalence of colonization including methicillin-resistant Staphylococcus aureus, vancomycin-resistant Enterococcus spp.	Further deterioration of renal function and even renal failure	Zacharioudakis et al., 2015; Wang et al., 2019
	The expression of genes related to the TMA metabolic pathway was altered	Cardiovascular risk increases	Xu et al., 2017
CVD	Enterobacteriaceae, oral resident bacteria, butyric acid-producing bacteria were abundant	Development of CVD	Jie et al., 2017
	α-diversity of gut ARG were positively correlated with triglycerides and total cholesterol/HDL, but negatively associated with HDL cholesterol	High risk of CVD	Shuai et al., 2022
	Prolonged antibiotic use	High future CVD incidence	Yuan et al., 2020
ASD	Three ARGs, aac(6′)-aph(2″), cepA-49, and tet(40) increased	Cause neurodevelopmental disorders	Kovtun et al., 2020
IBD	Abundance of Escherichia coli increased, multiple ARGs increased, such as mdtO, mdtP, emrK	Accelerate IBD progression	Xia et al., 2021
	Beta-lactam, fosmidomycin, multidrug and polymyxin resistance genes enriched in CD	Pathogenic bacteria increased	Xia et al., 2021

## Current therapeutic situation for antibiotic resistance genes

### Rifaximin positively regulates gut microbiota antibiotic resistance genes

Exposure to ciprofloxacin, amoxicillin, and metronidazole, beyond 5 days, is associated with a marked increase in ARG abundance (Bajaj et al., 2021). Rifaximin treatment, on the other hand, is positively correlated with the abundance of beneficial taxa and negatively correlated with the abundance of Klebsiella spp. resistome and Gram-negative ARGs. Rifaximin is a derivative of rifamycin, which is produced by Streptomyces spp. Interestingly, the Streptomyces spp. resistome increases in cirrhosis patients compared with diabetes or CKD patients (Bass et al., 2010; Shamsaddini et al., 2021). Encouraging the use of non-absorbable enteric-specific antibiotics, such as rifaximin, can improve the prognosis of cirrhosis patients.

### Microbiota-based therapy

Gut microbiota are recognized as a potential reservoir for the transmission of drug-resistant genes from symbionts to pathogens. Microbiota-based therapies can reduce the abundance of ARGs and ARBs in the gut microbiome of patients, reduce their risk of ARB-induced infections, and reduce the risk of transfer of pathogens carrying ARGs to other individuals (Langdon et al., 2021). To restore the biodiversity of microbial communities, probiotics can be used to replace important missing or depleted species or strains (Blaser, 2016). Probiotics impact the ARG reservoir in the human gastrointestinal microbiota in an antibiotic-dependent way. Montassier et al. (2021) showed that disruption of the gut microbiota by antibiotics supports probiotic colonization, but that the probiotics can amplify strains carrying vancomycin resistance genes, aggravating the amplification of the resistome in the gastrointestinal mucosa, and may themselves be reservoirs for the amplification of resistome in the gut. Thus, the potential transfer of ARGs through probiotics in the human intestine needs to be further evaluated.

### Fecal microbiota transplantation

ARGs associated with poor outcomes can be used as prognostic predictors and new therapeutic strategies could be developed to target these ARGs. Fecal microbiota transplant (FMT) is one such strategy and is associated with a decline in the abundance of ARGs (Saha et al., 2019; Bajaj et al., 2021). Metagenomic analysis revealed that FMT can reduce the burden of ARG in both cirrhosis and non-cirrhosis patients and the expression of gut ARGs was reduced in patients with decompensated cirrhosis after receiving capsule or enema FMT. After capsule FMT treatment patients showed a reduction in abundance of vancomycin (VanH), beta-lactamase (ACT), and rifamycin ARGs. After receiving antibiotics and enema FMT, vancomycin and beta-lactamase ARGs declined by day 15 and the abundance of cephalosporinase cepA, Enterococcus faecalis vanW, lincosamides (including clindamycin), and aminocoumarin resistance genes was also reduced. Irrespective of the mode of FMT dosing, significantly lower abundance of ARGs was observed in the decompensated cirrhosis group than in the pre-FMT and non-FMT groups. Moreover, vanH abundance increased, with time, in the placebo group but this was not found in the FMT group, vanH enhances vancomycin resistance of the cell wall of E. faecium (Bajaj et al., 2021). Thus, the beneficial effect of FMT in reducing the abundance of pathogen-related ARGs, seen for other diseases in previous studies, can now be extended to the field of advanced cirrhosis. Monitoring ARGs should not only improve prognosis, but will also allow the selection of appropriate treatment options for more abundant ARGs. However, extensive trials are still needed to verify that FMT reduces antibiotic resistance in patients with cirrhosis. Targeted intervention of specific microbiomes through FMT is also a potential strategy for the treatment of CKD (Meijers et al., 2019).

## Prospection

The amount, characteristic, and function of ARGs, collectively called the resistome, is the sum of all ARGs and their precursors in the microbiota (D’Costa et al., 2006; Wright, 2007; Casals-Pascual et al., 2018; Schwartz et al., 2020). The next generation of resistome research promises to combat emerging resistance threats (Crofts et al., 2017). We conclude that the composition of the gut microbiota is altered after antibiotic therapy with increased abundance of ARB, which further increases the abundance of ARGs in the gut. While some of the ARB in the gut microbiota were replaced by normal flora after FMT which induces a reduction in the abundance of ARGs. In addition, the individual baseline of gut microbiome can influence resistome alterations. When individual baseline species diversity is high, patients are more likely to experience an increase in ARGs during antibiotic treatment (Willmann et al., 2019). To better predict the impact of individual antibiotic on the gut microbiome and resisitome, further studies focused on the individual baseline of gut microbiome are needed. Antibiotic use has an impact on both gut microbiota and ARGs and identifying disease-host-specific ARGs should provide tailored antibiotic treatment strategies for the clinic. For example, the cfxA gene is significantly enriched in the gut of patients receiving antibiotic therapy in 1 month and is considered a potential biomarker to differentiate between patients and healthy populations (Duan et al., 2020). To date, few studies have evaluated the monitoring and management of ARGs. Thus, more research is needed to confirm the effectiveness of treatment against gut-specific ARGs, in different disease states, and the link between gut ARGs and fecal metabolites. Some studies have already found an association between ARGs and chronic liver disease, diabetes, CKD and CVD, but more research is needed to confirm the effect of ARGs on disease progression in these chronic diseases and to explore whether ARGs are altered in other chronic diseases such as chronic neurological disease and chronic respiratory disease. Monitoring ARGs is particularly important in the chronic disease management (CDM), in order to minimize damage to essential commensal microorganisms, individualized treatment strategies targeting gut-specific ARGs are expected to be a new direction in the future CDM.

## Concluding remarks

The presence of ARGs in the gut microbiota underpins the increasing failure of treatments for fatal bacterial infections. Since the misuse of antibiotics aggravates the production and spread of ARGs, limiting antibiotic overuse is an important tool to alleviate antibiotic resistance. A better awareness of HGT in the gut will help to open up new and effective interventions to reduce the transmission of ARGs. Altered gut microbiota and characteristic ARGs are strongly associated with disease progression in a number of chronic diseases. Future research is needed to confirm the effects of ARGs on the gut microbiota in patients with such diseases. Novel ARGs are still emerging and the detection and targeting of ARGs, as treatment goals, can change the prognosis of chronic diseases. Aiming to reduce the abundance of intestinal ARBs and slow down pathologic progression in chronic diseases, the individualized therapies focus on intestinal ARGs require more attention and further research.

## Author contributions

XP carried out the initial literature review and wrote the initial manuscript. ZZ have revised the manuscript. BL have collected the relevant literature data. ZW have provided expertise and insight relating to ARGs and checked the manuscript. All authors have read and approved the final manuscript.
